# Functional isogenic modeling of *BRCA1* alleles reveals distinct carrier phenotypes

**DOI:** 10.18632/oncotarget.4595

**Published:** 2015-06-23

**Authors:** Rory L. Cochran, Justin Cidado, Minsoo Kim, Daniel J. Zabransky, Sarah Croessmann, David Chu, Hong Yuen Wong, Julia A. Beaver, Karen Cravero, Bracha Erlanger, Heather Parsons, Christopher M. Heaphy, Alan K. Meeker, Josh Lauring, Ben Ho Park

**Affiliations:** ^1^ The Sidney Kimmel Comprehensive Cancer Center, The Johns Hopkins University School of Medicine, Baltimore, MD, USA; ^2^ The Whiting School of Engineering, Department of Chemical and Biomolecular Engineering, The Johns Hopkins University, Baltimore, MD, USA; ^3^ Oncology iMED, AstraZeneca, Waltham, MA, USA

**Keywords:** BRCA1, VUS, breast cancer, haploinsufficiency, centrosome amplification

## Abstract

Clinical genetic testing of *BRCA1* and *BRCA2* is commonly performed to identify specific individuals at risk for breast and ovarian cancers who may benefit from prophylactic therapeutic interventions. Unfortunately, it is evident that deleterious *BRCA1* alleles demonstrate variable penetrance and that many *BRCA1* variants of unknown significance (VUS) exist. In order to further refine hereditary risks that may be associated with specific *BRCA1* alleles, we performed gene targeting to establish an isogenic panel of immortalized human breast epithelial cells harboring eight clinically relevant *BRCA1* alleles. Interestingly, *BRCA1* mutations and VUS had distinct, quantifiable phenotypes relative to isogenic parental *BRCA1* wild type cells and controls. Heterozygous cells with known deleterious *BRCA1* mutations (185delAG, C61G and R71G) demonstrated consistent phenotypes in radiation sensitivity and genomic instability assays, but showed variability in other assays. Heterozygous *BRCA1* VUS cells also demonstrated assay variability, with some VUS demonstrating phenotypes more consistent with deleterious alleles. Taken together, our data suggest that *BRCA1* deleterious mutations and VUS can differ in their range of tested phenotypes, suggesting they might impart varying degrees of risk. These results demonstrate that functional isogenic modeling of *BRCA1* alleles could aid in classifying *BRCA1* mutations and VUS, and determining *BRCA* allele cancer risk.

## INTRODUCTION

Inheritance of a deleterious mutation in either of the *BRCA1* or *BRCA2* genes greatly increases a woman's lifetime risk of developing breast and ovarian cancers [1, 2]. As a result, clinical *BRCA* germline DNA testing has become routine for high risk patients and family members likely to benefit from preventative strategies [3]. Unfortunately, in addition to revealing benign and disease causing alleles, genotyping has also uncovered nearly 2,000 distinct *BRCA* alleles of unclear clinical significance [4]. These variants of unknown significance (VUS), comprised mainly of missense, intronic and regulatory region variants, present a major problem for cancer prevention efforts and have led to many studies aimed at determining their role in disease [5, 6]. Notably, cell-based functional analyses of *BRCA1* variants have been particularly useful for both classification studies and understanding *BRCA1*'s role in tumorigenesis [6-8].

Accumulating evidence suggests that the *BRCA* genes may be distinct from other tumor suppressors, in that *BRCA* mediated tumorigenesis may not follow the proposed two-hit hypothesis [9]. The earliest indication for this notion came from mouse knock-out studies demonstrating that homozygous germ-line disruption of *BRCA1* led to embryonic lethality; however, lethality could be rescued by prerequisite mutations that were permissive for *BRCA1* bi-allelic loss [10-12]. Our lab previously performed somatic cell gene targeting to introduce a common *BRCA1* deleterious mutation, 185delAG, in two distinct non-tumorigenic human breast epithelial cell lines to create isogenic models of the heterozygous carrier state [13]. Using several different functional assays, we found that heterozygous *BRCA1*^185delAG^ cells manifested phenotypes previously associated with *BRCA1* null cancer cells, including decreased proliferation, altered cell-cycle profiles, increased sensitivity to ɣ-irradiation and increased genomic instability. These results support the hypothesis that human breast cells hemizygous for *BRCA1* are haploinsufficient, which presumably accelerates tumorigenesis in carriers with deleterious mutations [14]. Other groups have provided corroborating evidence that hemizygous *BRCA1* cells also display alterations in genomic integrity as well as regulation of centrosome duplication [15, 16]. In addition, recent evidence suggests that heterozygous *BRCA1* mutant cells have an exaggerated response to estrogen induced DNA damage, providing a possible explanation of the cancer tissue specificity seen in *BRCA* carriers [16].

We reasoned that we might be able to extend our isogenic model to determine the functional consequences of *BRCA1* VUS, and therefore aid in risk assessment. Additionally, this approach could theoretically detect phenotypic differences between distinct deleterious alleles. As *BRCA1* is involved in multiple cellular processes, it is likely that deleterious mutations are not all equivalent, and therefore would have varying relative risks for cancer development. Although prior models examining *BRCA* alleles have been published [8, 17-20], to our knowledge, no studies have utilized gene targeting/genome editing of endogenous alleles within a non-tumorigenic human breast epithelial cell line to precisely recapitulate the carrier state. This approach generates a panel of isogenic cell lines to evaluate *BRCA1* mutations versus the parental wild type *BRCA1* cell line, and also allows for comparison between distinct *BRCA1* mutations and VUS. Here, we used adeno-associated viral (AAV) mediated gene targeting to genetically engineer an isogenic panel of heterozygous *BRCA1* mutations/VUS within the non-tumorigenic human breast epithelial cell line, MCF-10A. Using functional assays including proliferation rate, ɣ-irradiation sensitivity and genomic instability, we demonstrate that *BRCA1* VUS/mutations have a spectrum of phenotypes, suggesting that both deleterious mutations and VUS impart relative degrees of risk for cancer development.

## RESULTS

### Engineering an isogenic *BRCA1* cell line panel

**Figure 1:**
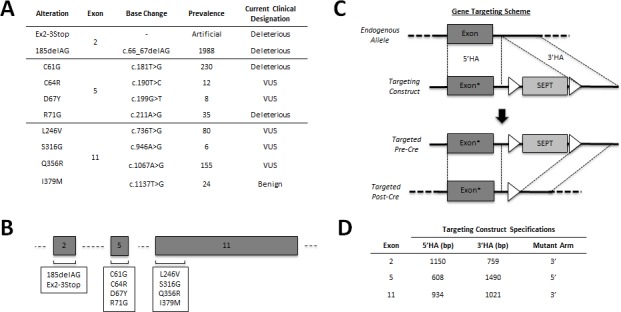
Engineering an isogenic BRCA1 panel Overview of the *BRCA1* alleles genetically engineered in this study. **A.** Table showing genomic locus, prevalence and current designation for each introduced allele. The prevalence represents the number of unique entries cataloged into the Breast Cancer Information Core (BIC), as of January 2014. The current clinical classification of each allele is also taken directly from the BIC. **B.** Schematic representation of each exon targeted in this study (not drawn to scale). **C.** Representative rAAV-mediated gene targeting schema for a targeting vector with an exonic mutation (denoted by the asterisk) within the 5′ homology arm (HA). First, rAAV transduction facilitates integration of the targeting vector via homologous recombination between the 5′ and 3′HAs. Following neomycin selection and clone isolation, the *loxP* flanked (white triangles) SEPT cassette is excised using Cre recombinase, leaving only a small intronic *loxP* scar. **D.** Table showing both the homology arm sizes and the respective mutant arm for each of the gene targeting constructs used in this study. MCF-10A gDNA was used as PCR template for each *BRCA1* targeting construct.

To perform *in vitro* modeling of different *BRCA1* carrier states, gene targeting experiments were conducted in the human non-tumorigenic breast epithelial cell line, MCF-10A (14). AAV targeting vectors were used to generate at least two independent clones for each mutation/VUS, as described in Methods. The panel members described herein include our previously characterized *BRCA1*^185delAG^ clones, a pair of newly engineered hemizygous *BRCA1* knock-out clones (*BRCA1*^Ex2-3Stop^) and eight sets of missense mutations including two deleterious alleles (C61G and R71G), five VUS (C64R, D67Y, L246V, S316G and Q356R) and one benign variant (I379M). To control for clonal variation and/or possible effects caused by the gene targeting process, three independent targeted wild-type clones (two for exon 5 and one for exon 11) were established and used in parallel for all assays. Figure 1A lists each of the engineered *BRCA1* alleles, their recorded prevalence, and clinical designation. All cell lines were verified to have a single integrated copy of the desired mutation and allelic expression equivalent to the wild type allele using PCR and RT-PCR followed by Sanger sequencing (Figure S1).

### Distinct *BRCA1* mutations and VUS can decrease cell proliferation

**Figure 2:**
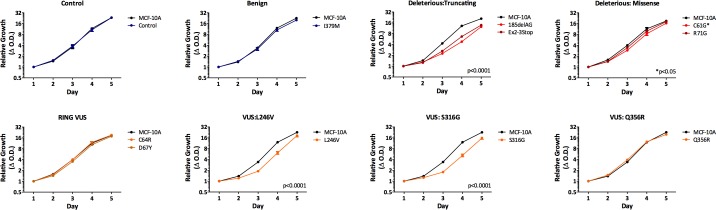
Cell proliferation Mean relative proliferation rates for all isogenic panel members, as measured by the SRB assay (error = SEM). Values represent means across at least two clones per mutation with each assayed at least in triplicate. Clones demonstrating significantly different growth rates are indicated.

We initially chose to assess the proliferation rates for each *BRCA1* mutation/VUS cell line, since we previously described that cells harboring the *BRCA1*^185delAG^ allele demonstrated reduced proliferation compared to parental MCF-10A cells. As shown in Figure 2, cells harboring a truncating allele, either *BRCA1*^Ex2-3Stop^ or *BRCA1*^185delAG^ cells, showed significantly decreased proliferation compared to parental MCF-10A and control cells. In contrast, cells harboring the deleterious missense mutation *BRCA1*^R71G^ grew similar to controls, with *BRCA1*^C61G^ deleterious missense mutant clones exhibiting an intermediate change in proliferation. These results suggest that mutations classified as deleterious, are not functionally identical. Interestingly, our VUS panel also showed varying phenotypes. *BRCA1*^C64R^*, BRCA1*^D67Y^*, BRCA1*^Q356R^ cell lines did not appear to have significant changes in proliferation, whereas clones with *BRCA1*^L246V^and *BRCA1*^S316G^ mutations demonstrated decreased cell proliferation, suggesting that the *BRCA1*^L246V^and *BRCA1*^S316G^ VUS may have an adverse effect on normal BRCA1 function. Lastly, the *BRCA1*^I379M^ benign variant cells grew similar to both parental MCF-10A and its targeted wild-type control.

### Specific *BRCA1* genotypes confer sensitivity to ɣ-irradiation

**Figure 3:**
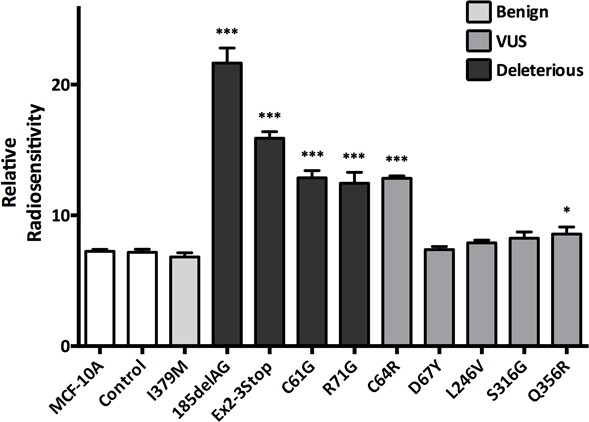
γ-Irradiation sensitivity Histogram showing mean relative radio-sensitivity (error = SEM) for at least two clones per mutation with each assayed at least in triplicate. Derivatives demonstrating statistically significant increases in radio-sensitivity, relative to MCF-10A and controls, are indicated.

Next, we assayed each cell line's sensitivity to ɣ-irradiation. As shown in Figure 3, cells harboring one truncating *BRCA1* allele demonstrated a statistically significant increase in sensitivity to ionizing radiation, consistent with our prior studies [13]. Notably, the *BRCA1*^185delAG^ cells demonstrated the largest radio-sensitivity across all deleterious clones, suggesting this mutation is distinct from other *BRCA1* mutations for this particular phenotype. In addition, the *BRCA1*^C61G^, *BRCA1*^R71G^, *BRCA1*^C64R^ and *BRCA1*^Q356R^ clones also demonstrated increased sensitivity to ionizing radiation. Similar to the relative change in proliferation rates, missense deleterious clones demonstrated a milder phenotype of ɣ-irradiation sensitivity compared to deleterious truncating clones, again suggesting a spectrum of functional deficits among deleterious alleles. The *BRCA1*^C64R^ VUS demonstrated increased radio-sensitivity similar to the *BRCA1*^C61G^ clones, suggesting this variant may have deleterious properties. Interestingly, the *BRCA1*^Q356R^ VUS also demonstrated a small increase in radio-sensitivity. In contrast, cells harboring the *BRCA1*^I379M^ did not display increased radio-sensitivity compared to controls, consistent with its designation as a benign variant.

### *BRCA1* variants can induce genomic instability

**Figure 4:**
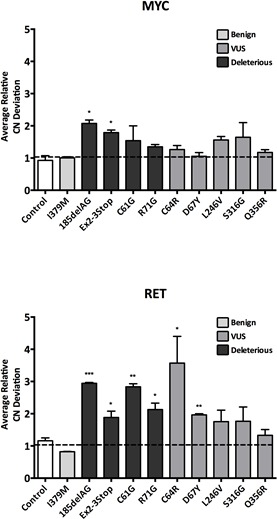
Chromosomal Instability (CIN) CIN as measured by fluorescent *in situ* hybridization (FISH). To control for experimental variability across multiple experiments, each independent experiment included parental MCF-10A alongside each engineered derivative. Therefore all data is reported relative to parental MCF-10A. Histograms display average copy number (CN) deviations for all panel members using the *MYC* and *RET* gene probes (error = SEM). Derivatives demonstrating statistically significant differences are indicated.

To assess chromosomal instability (CIN), we chose to use fluorescence *in situ* hybridization (FISH). FISH can assess the genomic state (i.e. ploidy) of a cell population, and can also analyze a cell population's rate of chromosomal instability (CIN), defined as the frequency of copy number deviation from the mode across many cells within a given cell population [21]. For all isogenic cell lines, we evaluated CIN using two gene probes (*MYC* and *RET*). CIN estimates are reported as the average relative copy number deviation for each probe as shown in Figure 4. As expected, cells harboring a truncating *BRCA1* allele demonstrated an increase in CIN for both gene probes tested. Interestingly, the two sets of missense deleterious clones, *BRCA1*^C61G^ and *BRCA1*^R71G^, also demonstrated CIN, but only for the *RET* gene probe. This could be due to either the sensitivity of different FISH probes for measuring CIN and/or the possibility that certain deleterious mutations may have a more pronounced effect on CIN. Nonetheless, these results further demonstrate that not all deleterious *BRCA1* mutations are functionally equivalent.

The *BRCA1*^C64R^ and *BRCA1*^D67Y^ VUS were the only VUS derivatives demonstrating statistically significant CIN. Consistent with the irradiation experiments, these results suggest that the *BRCA1*^C64R^ VUS has deleterious properties. Although the *BRCA1*^D67Y^ cells did demonstrate statistically significant CIN, the difference from controls was relatively small. In accord with other work, these results suggest that the *BRCA1*^D67Y^ allele may be hypomorphic [8, 22]. As expected, cells harboring the *BRCA1*^I379M^ benign variant did not demonstrate CIN, further corroborating its neutral effect on protein function.

### Cell-cycle analysis reveals an increased fraction of polyploid cells in *BRCA1* mutant/VUS clones

**Figure 5:**
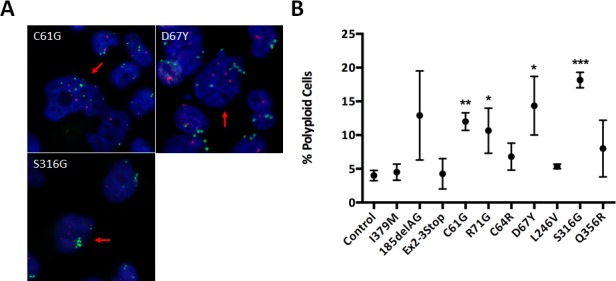
Polyploidy estimation **A.** Representative FISH images for derivatives demonstrating large abnormally shaped nuclei with >2x the modal copy number for *MYC* and/or *RET* genes, indicated by the red arrows. Green foci indicate *MYC* alleles and red foci indicate *RET* alleles, with the nucleus shown in blue (DAPI). **B.** Scatter plot showing the percent observed polyploidies relative to parental MCF-10A (error = SEM), across at least two clones for each. Derivatives demonstrating statistically significant increases are indicated.

When analyzing *BRCA1* clones by FISH, we also observed that many of the clones demonstrating CIN appeared to display a small but notable fraction of cells with >4n for one or both gene probes (Figure 5A). These observations prompted us to perform cell cycle analysis for ploidy estimation. Interestingly, the deleterious missense mutants, *BRCA1*^C61G^ and *BRCA1*^R71G^, demonstrated a larger fraction of polyploid cells, compared to control cells, though some mutations did not demonstrate appreciable differences (Figure 5B). Again, these results suggest that not all deleterious mutations are functionally identical. Additionally, two VUS, *BRCA1*^D67Y^ and *BRCA1*^S316G^, demonstrated a statistically significant increase in polyploid cell fractions. Collectively, our results suggest that the *BRCA1*^D67Y^ VUS does abrogate normal BRCA1 function. As expected, the *BRCA1*^I379M^ clones demonstrated results similar to controls, suggesting this neutral variant has no impact on normal BRCA1 function.

### *BRCA1* mutants and VUS affect centrosome amplification

BRCA1 has been implicated in the regulation of centrosome duplication, whereby inhibition of BRCA1 or loss of its function leads to an increase in centrosome number [23]. Interestingly, a recent study showed that normal breast tissue from deleterious *BRCA1* carriers displayed increased centrosome number, relative to controls [15]. The abnormal centrosome dynamics observed in BRCA1 mutant cells appears to be due to alterations in BRCA1's RING domain, where the *BRCA1*^C61G^ and *BRCA1*^C64R^ mutations localize [8, 23, 24].

**Figure 6:**
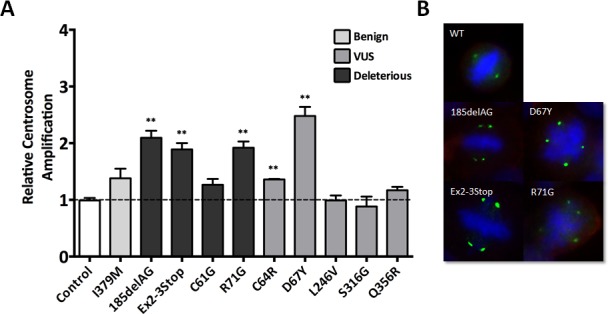
Centrosome amplification **A.** Average centrosome number relative to parental MCF-10A for all panel members (error = SEM), with derivatives demonstrating statistically significant amplification indicated. Similar to the FISH analyses described above, each independent experiment included parental MCF-10A alongside each engineered derivative, with data reported as relative to parental MCF-10A. **B.** Representative images for cells demonstrating multipolar mitoses due to aberrant centrosome number. Green represents immunofluorescent staining of ɣ-tubulin, blue identifies nucleic acids (DAPI) and red identifies the plasma membrane.

Therefore, to study whether our isogenic panel of cell lines also demonstrated an increase in overall centrosome number, we performed immunofluorescent labeling for ɣ-tubulin, a major centrosomal core protein, to quantify cellular centrosomes. As shown in Figure 6, and consistent with the studies of Martins et al. [15], we observed an increase in centrosome number for cells harboring a deleterious allele, namely the *BRCA1*^185delAG^, *BRCA1*^exon 2-3stop^ and *BRCA1*^R71G^ derivatives. Surprisingly, the *BRCA1*^C61G^ cells did not demonstrate a significant increase in centrosome number relative to parental MCF-10A or controls, and similarly, the *BRCA1*^C64R^ variant demonstrated only a slight increase in centrosome number. Nonetheless, the slight increase and consistent results with the *BRCA1*^C64G^ clones in other functional assays further suggests that the *BRCA1*^C64R^ variant is deleterious. Interestingly, the *BRCA1*^D67Y^ variant demonstrated the highest number of multi-polar mitoses among all cell lines, again suggesting this variant does affect normal BRCA1 function. Table 1 summarizes the results across all of the functional assays described above.

**Table 1:** Isogenic Panel Phenotype Summary

ID	Radiosensitive	Chromosomal Instability		Increased Polyploid Fraction	Centrosome Amplification	Current Clinical Designation
		MYC	RET			
Ex2-3Stop	+	+	+	−	+	N/A
185delAG	+	+	+	+	+	Deleterious
R71G	+	−	+	+	+	Deleterious
C61G	+	−	+	+	−	Deleterious
C64R	+	−	+	−	+	VUS
D67Y	−	−	+	+	+	VUS
S316G	−	−	−	+	−	VUS
Q356R	+	−	−	−	−	VUS
L246V	−	−	−	−	−	VUS
I379M	−	−	−	−	−	Benign

## DISCUSSION

*BRCA* VUS pose a formidable challenge for cancer prevention strategies. Approximately half of the cataloged *BRCA* alleles in the Breast Cancer Information Core (BIC) are currently designated as VUS. To address this ongoing problem, many studies have been performed in attempt to classify VUS [6, 7, 25, 26]. Arguably the most effective approaches utilize a number of clinically relevant features to calculate the probability that a given VUS is deleterious. Often, however, the number of reported carriers for a given VUS is small, sometimes consisting of only one individual. These small sample sizes, coupled with the incomplete penetrance of *BRCA-*linked disease and the high incidence of sporadic breast cancer, leads to imprecise statistical predictions of risk. Therefore, investigators have turned to additional approaches, including *in silico* analysis and direct functional testing.

Our isogenic genome editing of *BRCA1* alleles has several advantages that make it an attractive alternative to prior *BRCA1* modeling approaches. First, isogenic modeling in non-cancerous human breast epithelial cells permits investigators to evaluate BRCA1 function within the appropriate cellular context. In contrast, prior studies have studied BRCA1 function in either human breast *cancer* cell lines or mouse embryonic stem cells [6, 8, 17, 18]. Second, isogenic modeling facilitates the investigation of *BRCA1* at appropriate gene dosages and physiologic expression, under the control of the endogenous transcriptional and splicing machinery. The importance of controlling for these variables is demonstrated here with the study of the R71G splice site mutation [8, 19]. Third, since isogenic cell lines can be created in distinct genetically stable cell lines, it permits the interrogation of *BRCA1* function under tissue specific and genetically defined conditions, thereby potentially providing new insight into the effects of additional genetic modifiers. Fourth, this methodology also possesses the advantage of expandability. As a clearer understanding of BRCA1 function evolves, additional functional assays may be adapted for *in vitro* study. The experiments performed in this study were chosen because they provided a time efficient set of assays that allowed us to maximally query for non-overlapping phenotypes, which may be desirable for future clinical use of this technology. Lastly, isogenic modeling of distinct deleterious alleles is ideal for drug screens aimed at overcoming some of the deficiencies associated with BRCA1 haploinsufficiency, such as restoration of homologous recombination through inhibition of 53BP1 [27].

We used isogenic modeling to evaluate functional differences between nine clinically relevant *BRCA1* alleles, including three deleterious, five VUS and one benign variant. Several important conclusions are made from the experiments described. Significantly, our data suggests that distinct deleterious *BRCA1* mutations confer varying degrees of functional loss *in vitro*. Notably, truncating *BRCA1* mutations lead to a higher level of sensitivity to ionizing radiation and genomic instability, as compared to other cancer associated *BRCA1* missense mutations. Whether these functional differences can truly influence disease penetrance remains to be determined, but we envision that the evidence presented here could be incorporated towards risk assessment in future studies and models. In addition, and of equal importance, our data suggest isogenic cell modeling may be useful for VUS classification. As the most notable example, characterization of the C64R VUS suggests this variant is functionally similar to the C61G deleterious mutation, since cells harboring this mutation were sensitive to ionizing radiation, and demonstrated increased genomic instability and centrosome amplification. Furthermore, functional characterization of the D67Y mutation suggests it may be hypomorphic despite prior work suggesting it is benign [28]. Similarly, cells harboring the S316G and Q356R were deficient in only a single functional assay, suggesting both may have hypomorphic properties. Despite the suppressed cell proliferation observed for cells harboring the L246V VUS, this variant did not demonstrate an appreciable difference from controls in the other assays, suggesting this variant may be benign. Nonetheless, further work is needed to determine if our *in vitro* model accurately reflects *in vivo* assays, and ultimately relative disease risk.

As with all models, the approach taken here has caveats. Unlike our prior study, only one breast cell line, MCF-10A, was used to generate our *BRCA1* panel. While this has the advantage of having an entire isogenic panel to compare between *BRCA1* mutations and VUS, it is possible that pre-existing genetic modifiers within the MCF-10A genome can influence the effects observed for a given engineered mutation. Although we have used other non-tumorigenic breast epithelial cell lines in the past [13], the immortalized hTERT cell line (hTERT-IMEC) has been shown to develop TP53 mutations with longer term culture [29], making it less ideal for these studies. In addition, the hTERT-IMEC AAV gene targeting rates are significantly lower compared to MCF-10A cells (unpublished results), hindering the ability to create a large isogenic panel. Future studies could rely upon the use of new culturing techniques [30] as well as improved genome editing tools such as CRISPR/Cas systems [31] to circumvent these limitations. Finally, the number of deleterious and benign mutations engineered in this study is relatively small, and therefore we cannot make broader conclusions about the clinical applicability of isogenic modeling for *BRCA1* allele risk assessment. However, this study does provide a proof of principle and the foundation for future work that could ultimately translate our isogenic cell line panel towards clinical utility.

## MATERIALS AND METHODS

### Cell culture

The non-tumorigenic human breast epithelial cell line MCF-10A [32] was purchased from ATCC (Manassas, VA, USA), and along with isogenic derivatives were grown in DMEM/F12 (1:1) media supplemented with 5% horse serum (Life Technologies, Carlsbad, CA, USA), 20 ng/mL epidermal growth factor (Sigma-Aldrich, St. Louis, MO, USA), 10 μg/mL insulin (Life Technologies, Carlsbad, CA, USA), 0.5 μg/mL hydrocortisone (Sigma-Aldrich, St. Louis, MO, USA), 0.1 μg/mL cholera toxin (Sigma-Aldrich, St. Louis, MO, USA) and 1% Penicillin-Streptomycin (Life Technologies, Carlsbad, CA, USA). All cell lines were verified by STR profiling and tested for mycoplasma contamination. Cells were maintained in a 37°C incubator with 5% CO_2_.

### Gene targeting in MCF-10A cells

Details for each of the *BRCA1* alleles genetically engineered in this study are shown in Figure 1A. The *BRCA1* exon 2-3Stop mutation was engineered to knock out one allele of *BRCA1* by both deleting the splice acceptor of exon 2 and replacing the start ATG with three stop codons in every reading frame (TAGaTAAcTGA). The exon 2-3Stop cells and our previously described *BRCA1*^185delAG^ clones [13] were used as positive controls. All other gene targeting of the *BRCA1* gene was carried out using distinct recombinant AAV vectors for each of the three respective *BRCA1* exons shown in Figure 1B. Gene targeting was performed as previously described (schematically represented in Figure 1C) [33]. AAV targeting vectors were constructed by ligating wild type homology arms synthesized by PCR into an AAV plasmid backbone (Agilent, La Jolla, CA, USA). We then employed site-directed mutagenesis by overlap extension PCR [34] with subsequent cloning back into the parental AAV plasmid backbone, to generate each of the respective variant/mutant constructs listed in Figure 1A. PCR primers for homology arm construction are listed in Table S1. Mutagenesis primers used to create each of the missense mutations are listed in Table S2. Infectious virus was prepared by co-transfecting HEK-293T cells (ATCC, Manassas, VA, USA) with pHelper, pRC (Agilent, La Jolla, CA, USA) and the respective *BRCA1* rAAV gene targeting plasmid as previously described. Approximately 10^6^ MCF-10A cells were used for each viral infection, and a sib selection strategy was employed as previously described [35]. The ‘pre-Cre’ PCR screening primers are listed in Table S3. After isolation of targeted neomycin resistant clones, the cells were then exposed to Cre-expressing recombinant adenovirus to remove the neomycin cassette as previously described [33]. PCR screening primers are listed in Tables S3 and S4. All clones were subjected to confirmation by Sanger sequencing of genomic DNA and cDNA to ensure each clone was monoclonal and harbored the relevant *BRCA1* alterations as single expressed copies. Cell line genomic DNA (gDNA) was isolated using a QIAamp® DNA Blood Mini kit (Qiagen, Valencia, CA, USA). All conventional Sanger sequencing of genomic DNA was carried out following PCR amplification of respective loci using Phusion® High-Fidelity DNA Polymerase (New England BioLabs, Ipswich, MA, USA). Preparation of cDNA derived RNA was performed using a First-Strand cDNA Synthesis kit (GE Healthcare, Pittsburgh, PA, USA). Primers used for clone confirmation are shown in Tables S5–S8. At least two clones were isolated for each mutation. In addition, AAV gene targeting using wild type *BRCA1* constructs for both exon 5 and exon 11 was carried out to create targeted wild-type controls for each locus. In each of the described assays, the presented data represents means across ≥2 clones for each genetically distinct panel member. To control for clonal variation and/or possible effects caused by the gene targeting process, three independent targeted wild-type clones (two for exon 5 and one for exon 11) were established. All derivatives and control clones were assayed at similar passage number. For clarity, data generated for all three targeted wild-type control clones were grouped together.

### Cell proliferation assays

Relative proliferation rates were assessed after plating 2 × 10^3^ cells into 96-well plates and measuring total cellular protein using the sulforhodamine B (SRB) assay, as previously described [36]. All chemicals used for the SRB assay were purchased from Sigma-Aldrich (St. Louis, MO, USA).

### ɣ-Irradiation sensitivity assay

ɣ-irradiation sensitivity was determined as previously described [13]. Briefly, cells were sparsely seeded and treated with either zero or six Gy of radiation at a dose of approximately 3.63 Gy/minute using a Xstrahl X-Ray irradiator. Following treatment, cells were maintained for 8-10 days until colonies were visible. Cells were washed with PBS then fixed and stained with 3.7% formaldehyde containing 0.2% (wt/vol) Crystal violet (Sigma-Aldrich, St. Louis, MO, USA). Relative radio-sensitivity was defined as the inverse of fractional survival at 6 Gy.

### Fluorescent *in situ* hybridization (FISH) and CIN assays

FISH was performed as previously described [13]. Briefly, 10^5^ cells were plated in 8-well BD Falcon^TM^ glass chamber slides (Thermo Fisher Scientific, Waltham, MA, USA) and grown exponentially for two days before being fixed with 10% neutral buffered formalin (Sigma-Aldrich, St. Louis, MO, USA). Following fixation, cells were washed in phosphate buffered saline (PBS) pH 7.4 (Life Technologies, Carlsbad, CA, USA) and treated with a FISH pretreatment reagent kit (Abbott Molecular, Des Plaines, IL, USA), according to the manufacturer's recommendations. Pretreated, dehydrated slides were then probed simultaneously with Vysis LSI *RET* (Tel) SpectrumOrange and Vysis LSI *MYC* SpectrumGreen gene probes (Abbott Molecular, Des Plaines, IL, USA). The *RET* probe localizes to the 10q11.21 region and the *MYC* probe localizes to the 8q24 region. Following probe hybridization, slides were counterstained with 0.5 μg/mL 4,6-diamidino-2-phenylindole (DAPI) (Sigma-Aldrich, St. Louis, MO, USA) for 5 minutes and mounted with Prolong Gold (Life Technologies, Carlsbad, CA, USA). At least 200 interphase cells were counted to assess for gene copy number gains and losses. Percent copy number (CN) change from the mode was determined as the percentage of cells whose copy number deviated from the modal population for each gene probe.

### Polyploidy analysis

Polyploidy analysis was carried out by seeding 5.0 × 10^5^ cells and then culturing for two days. Cells were trypsinized, washed with cold PBS and fixed with 70% cold ethanol, then stored for 1-7 days at −20°C. Cells were then washed once with cold PBS before being stained with 40 μg/mL propidium iodide in the presence of 500 μg/mL DNase-free RNase A in 0.1% Triton X-100 in PBS at 37°C for 15 minutes, all from Sigma-Aldrich (St. Louis, MO, USA). For each isogenic derivative, at least 10^4^ cells were analyzed using a FACSCalibur™ (BD Biosciences) for cell cycle distribution and polyploidy (>4n). All data was normalized to parental MCF-10A and compared to targeted wild type controls.

### Immunofluorescence labeling

To assess centrosome number in MCF-10A cells and all engineered derivatives, 10^5^ cells were plated in chamber slides and grown for two days under exponential growth conditions, before being fixed in cold methanol for 15 minutes at −20°C. Following fixation, cells were washed once with cold acetone and treated for at least two hours with PBS containing 5% goat serum and 0.3% Triton X-100 (Sigma-Aldrich, St. Louis, MO, USA). A primary rabbit antibody against ɣ-tubulin (catalog# T5192, Sigma-Aldrich, St. Louis, MO, USA) was applied (at a 1:10^3^ dilution) for one hour followed by a 1:10^2^ dilution of secondary goat anti-rabbit antibody conjugated to Alexa Fluor 488 (catalog# A11034, Life Technologies, Carlsbad, CA, USA) for 20 minutes. The plasma membrane was stained with 5 μg/mL Texas Red^®^-X conjugated wheat germ agglutinin (WGA) for five minutes (Life Technologies, Carlsbad, CA, USA) and the nucleus was counterstained with 0.5 μg/mL 4′,6-diamidino-2-phenylindole (DAPI) for one minute (Sigma-Aldrich, St. Louis, MO, USA). The percentage of cells with greater than two centrosomes was determined by viewing at least 200 cells for each cell line.

### Statistical considerations

All statistical analyses were carried out using GraphPad Prism 6 software with *P* value significance levels indicated using one or more asterisks: *P* ≤ 0.05 (*), *P* ≤ 0.01 (**) and *P* ≤ 0.001 (***). Relative proliferation rates were analyzed by two-way ANOVA. Relative radio-sensitivities were compared to both parental MCF-10A and controls using unpaired t-tests. Results from the FISH, immunofluorescence and cell-cycle experiments were compared to control samples using unpaired t-tests.

## SUPPLEMENTRY MATERIAL FIGURES AND TABLES


