# Supplementary material for: Functional isogenic modeling of BRCA1 alleles reveals distinct carrier phenotypes
Source: Oncotarget. 2015 Jun 23;6(28):25240–51. doi: 10.18632/oncotarget.4595 (PMC4694828; doi:10.18632/oncotarget.4595)
Supplement: Supplementary file 1 [file oncotarget-06-25240-s001.pdf]

Functional isogenic modeling of *BRCA1* alleles reveals distinct carrier phenotypes

Supplementry Material

Table S1: Homology Arm Cloning Primers

Locus	Homology Arm	Forward/Reverse
Exon 2 (3Stop)	5’	5’-TCGTATTCTGAGAGGCTGCTGCT<br>5’-TCACATATTGCTGCCAACCC
	3’	5’-GCTCTTCGCGTTGAAGAAGTAC<br>5’-GCCAGTTTCAAACAAAGGTTCTTCC
Exon 5	5’	5’-CGCTTGATCACAGATGTATG<br>5’-CCAACCTAGCATCATTACCA
	3’	5’-AAGCAACCACAGTAGGAAAAAGTAG<br>5’-CTCGTACTTTCTTGTAGGCTC
Exon 11 (5’)	5’	5’-GAAATCAGTTTGGATTCTGCA<br>5’-TGGAAATCAACCAACTGGCT
	3’	5’-CCTCCAAGGTGTATGAAGTATG<br>5’-GGTTAGTTCCCTGATTTATCATTTC

Table S2: Mutagenesis Primers

Locus	Variant/Mutant	Forward/Reverse
Exon 5	C61G	5’-GGGCCTTCACAGgGTCCTTTATGTAAG<br>5’-CTTACATAAAGGACcCTGTGAAGGCCC
	C64R	5’-CAGTGTCCTTTACGTAAGAATGAT<br>5’-ATCATTCTTACGTAAAGGACACTG
	D67Y	5’-TATGTAAGAATtATATAACCAAAAGGT<br>5’-ACCTTTTGGTTATATaATTCTTACATA
	R71G	5’-GATATAACCAAAgGGTATATAATTTGG<br>5’-CCAAATTATATACCcTTTGGTTATATC
Exon 11 (5’)	L246V	5’-CAGTAATAATGATgTGAACACCACTGAG<br>5’-CTCAGTGGTGTTCAcATCATTATTACTG
	S316G	5’-CTTAGCAAGGgGCCAACATAAC<br>5’-GTTATGTTGGCcCCTTGCTAAG
	Q356R	5’-GAATGGAATAAGCgGAAACTGCCATGCTC<br>5’-GAGCATGGCAGTTTCcGCTTATTCCATTC
	I379M	5’-CTAAATAGCAGCATgCAGAAAGTTAATGAG<br>5’-CTCATTAACTTTCTGcATGCTGCTATTTAG

Table S3: Pre-Cre Screening Primers

Locus	Homology Arm	Forward/Reverse
Exon 2 (3Stop)	5’	5’-GACCTCTTCTTACGACTGCTTTG<br>5’-ATGTATGCTATACGAAGTTATGGATCC
	3’	5’-TTAAGGTACCACTGTGCATATG<br>5’-CCAGCCTCTCGACAGAGATC
Exon 5	5’	5’-GACCATCCTGGCTAACATGG<br>5’-CATTGTCACTCAAGTGTATGGC
	3’	5’-TTAAGGTACCACTGTGCATATG<br>5’-CTCCAAACCTGTGTCAAGCTG
Exon 11 (5’)	5’	5’-GACAGTTCTGCATACATGTAACTAG<br>5’-GCAGACAGCGAATTAATTCC
	3’	5’-TTAAGGTACCACTGTGCATATG<br>5’-CGAGTGATTCTATTGGGTTAGG

Table S4 Post-Cre Screening Primers

Locus	Forward/Reverse
Exon 2 (3Stop)	5’-GACCTCTTCTTACGACTGCTTTG<br>5’-CATATGCACAGTGGTACCTTAA
Exon 5	5’-ACTAGTGGATCCATAACTTCG<br>5’-CTCCAAACCTGTGTCAAGCTG
Exon 11 (5’)	5’-GACAGTTCTGCATACATGTAACTAG<br>5’-CATATGCACAGTGGTACCTTAA

Table S5: PCR Primers Across *loxP* Scar

Locus	Forward/Reverse
Exon 2 (3Stop)	5’-GGAGAAAGCTAAGGCTACC<br>5’-AGTGGATGGAGAACAAGGAA
Exon 5	5’-CTTAAGGGCAGTTGTGAGATTATC<br>5’-CCTGTATAAGGCAGATGTCC
Exon 11 (5’)	5’-CACTCTTAGACGTTAGAG<br>5’-CAAAAATAACAAGGTACTCAA

Table S6: Targeted Allele Sequencing Primers

Locus	Forward/Reverse	Nested
Exon 2 (3Stop)	5’-GACCTCTTCTTACGACTGCTTTG<br>5’-CATATGCACAGTGGTACCTTAA	5’-GGAGAAAGCTAAGGCTACC
Exon 5	5’-GACCATCCTGGCTAACATGG<br>5’-ATGTATGCTATACGAAGTTATGGATCC	5’-CTTAAGGGCAGTTGTGAGATTATC
Exon 11 (5’)	5’-TTAAGGTACCACTGTGCATATG<br>5’-CGAGTGATTCTATTGGGTTAGG	5’-TATATTTTCAGCTGCTTGTGAAT

Table S7: Bi-Allelic Sequencing Primers

Locus	Forward/Reverse	Nested
Exon 5	5’-ATTTCTGTACTGTCAATTCC<br>5’-CCAACCTAGCATCATTACCA	5’-CTTAAGGGCAGTTGTGAGATTATC
Exon 11 (5’)	5’-CCTCCAAGGTGTATGAAGTATG<br>5’-CCTAACAGTTCATCACTTCTGG	5’-TATATTTTCAGCTGCTTGTGAAT

Table S8: cDNA Sequencing Primers

Locus	Forward/Reverse	Nested
Exon 5	5’-AGCTCGCTGAGACTTCCTG<br>5’-GGAACATCTTCAGTATCTCTAGG	5’-GCCCATACTTTGGATGATAG
Exon 11 (5’)	5’-CCTTGGAACTGTGAGAACTCTG<br>5’-CGAGTGATTCTATTGGGTTAGG	5’-CTGCTTGTGAATTTTCTGAGAC

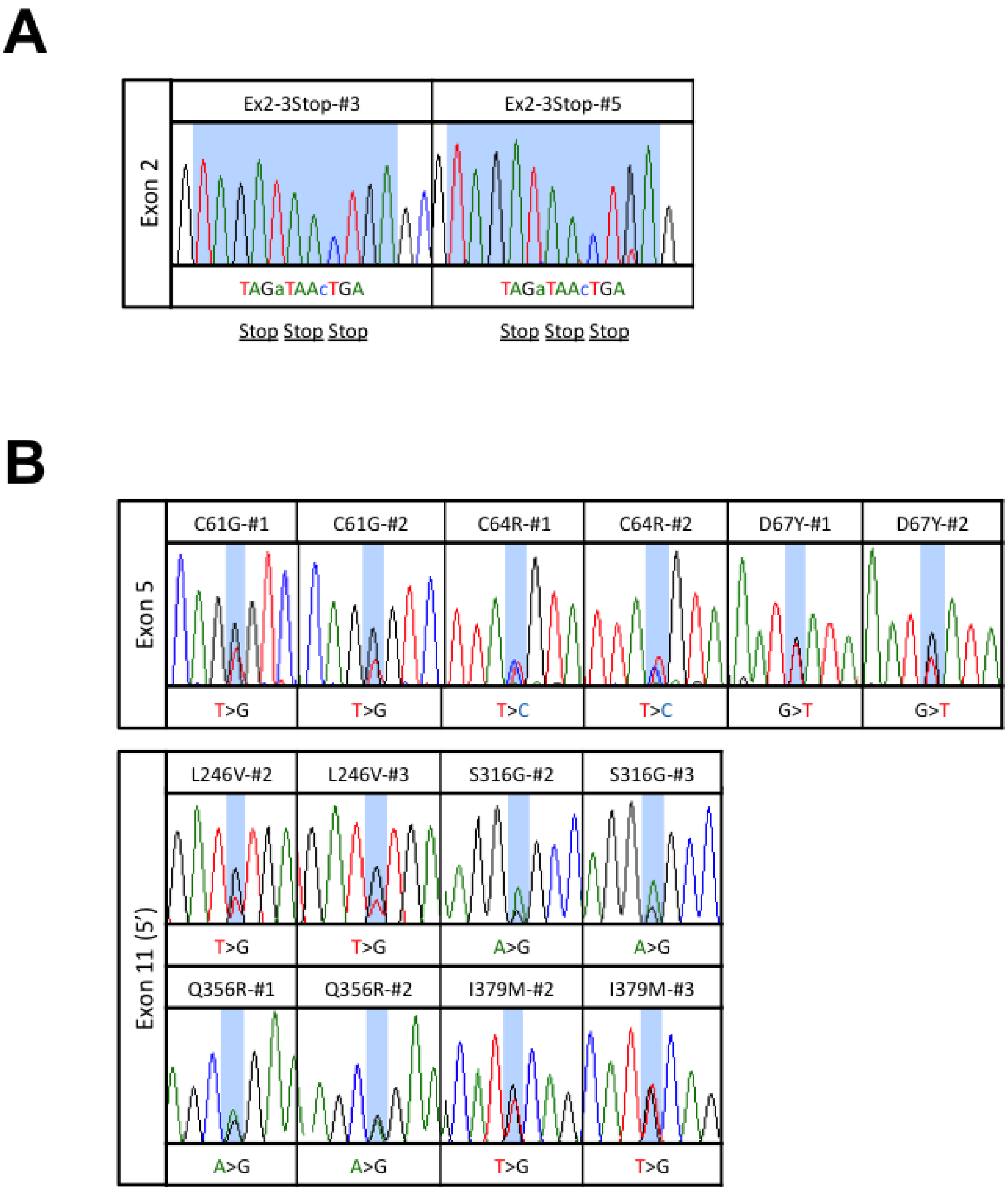

Figure S1: Sanger sequence traces for the engineered *BRCA1* panel members. A) gDNA sequences for the two exon 2-3stop hemizygous knock-out clones. B) cDNA sequences for the exon 5 and exon 11 clones. The R71G clones are not shown, as this mutation disrupts normal splicing.

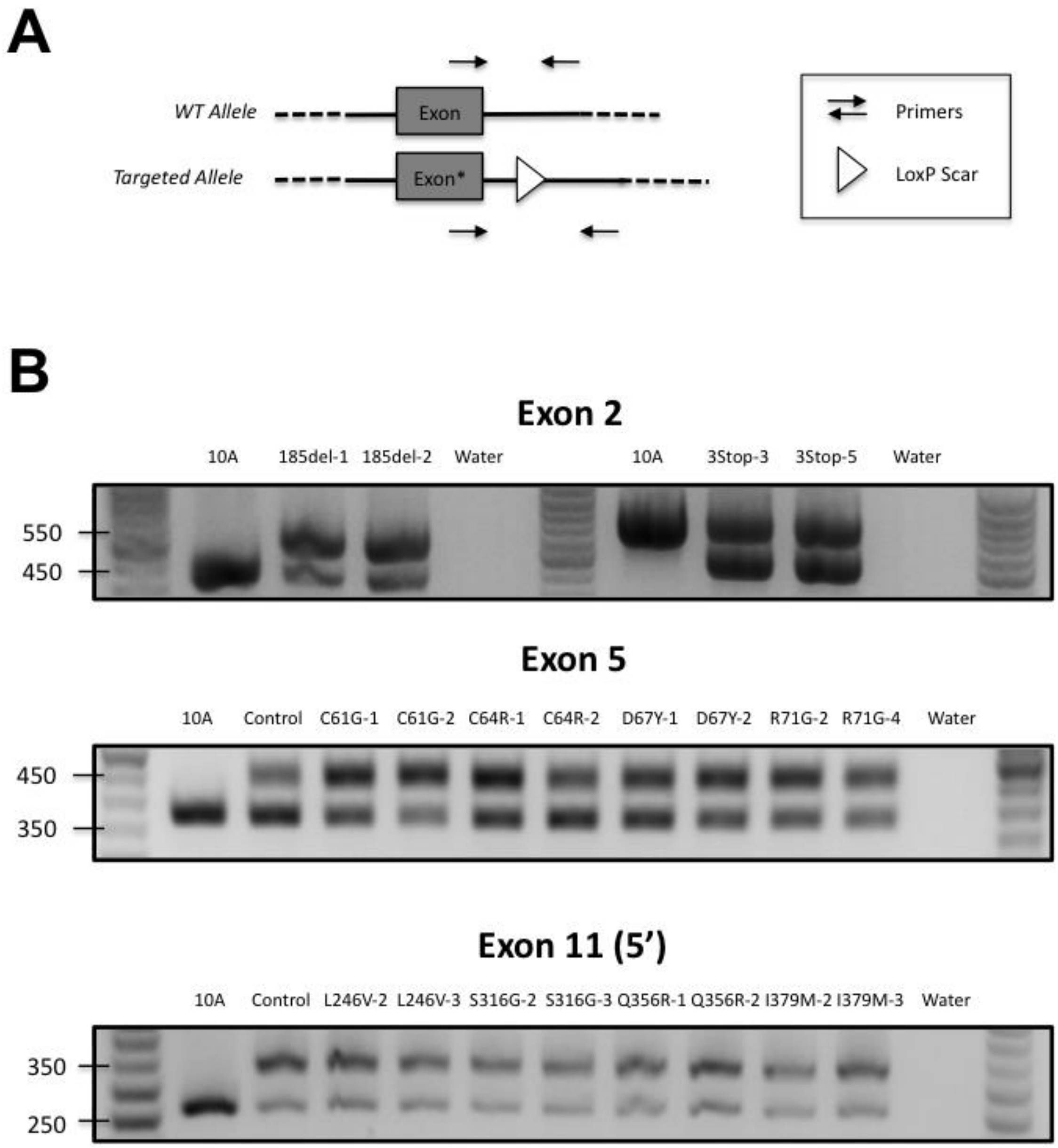

Figure S2: Genetic confirmation of isogenic panel members via PCR analysis. A) Schematic of PCR using loxP scar spanning genomic primers. As shown, PCR of the targeted allele generates a larger PCR amplicon. B) 2% agarose gel with PCR products for each of the three loci gene targeted.
